# 2-(4-Meth­oxy­benzyl­idene)-2*H*-1,3-benzodithiole 1,1,3,3-tetra­oxide

**DOI:** 10.1107/S1600536812002826

**Published:** 2012-02-04

**Authors:** Haruyasu Asahara, Peter Mayer, Herbert Mayr

**Affiliations:** a Ludwig-Maximilians-Universität, Department of Chemistry, Butenandtstrasse 5–13, 81377 München, Germany

## Abstract

The title compound, C_15_H_12_O_5_S_2_, crystallizes with two mol­ecules in the asymmetric unit. In both mol­ecules, the 1,3-benzodithiole plane and the aryl ring of the anisyl group are not quite coplanar; the corresponding dihedral angles are 20.4 (1) and 18.0 (1)°. π-Stacking [with centroid–centroid distances between 3.5440 (14) and 3.8421 (14) Å] takes place along [100] between the alternating benzodithiole benzene rings of symmetrically independent mol­ecules, and also between the anisyl groups of symmetrically related mol­ecules. Furthermore, mol­ecules are linked through C—H⋯O inter­actions.

## Related literature

For background on bis­ulfonyl ethyl­enes, see: Simpkins (1993 ▶); Najera & Yus (1999 ▶); Prilezhaeva (2000 ▶); Nielsen *et al.* (2010 ▶); Zhu & Lu (2009 ▶); Alba *et al.* (2010 ▶). For related structures, see: Giacometti *et al.* (1994 ▶); Zhang *et al.* (2010 ▶).
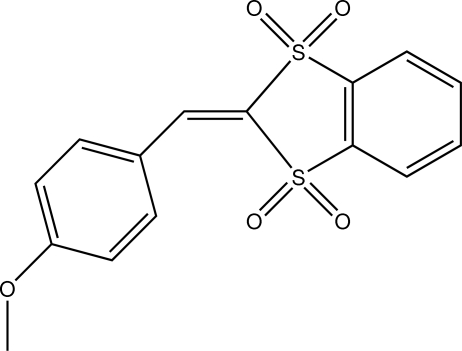


## Experimental

### 

#### Crystal data

C_15_H_12_O_5_S_2_
*M* *_r_* = 336.39
Triclinic, 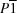

*a* = 7.3649 (2) Å
*b* = 11.4723 (3) Å
*c* = 17.6114 (5) Å
α = 84.345 (2)°
β = 84.631 (2)°
γ = 71.711 (2)°
*V* = 1402.89 (7) Å^3^
*Z* = 4
Mo *K*α radiation
μ = 0.40 mm^−1^
*T* = 173 K
0.17 × 0.12 × 0.08 mm

#### Data collection

Nonius KappaCCD diffractometer
9496 measured reflections
5124 independent reflections
4435 reflections with *I* > 2σ(*I*)
*R*_int_ = 0.019

#### Refinement

*R*[*F* ^2^ > 2σ(*F* ^2^)] = 0.037
*wR*(*F* ^2^) = 0.093
*S* = 1.10
5124 reflections
399 parameters
H-atom parameters constrained
Δρ_max_ = 0.29 e Å^−3^
Δρ_min_ = −0.38 e Å^−3^

### 

Data collection: *COLLECT* (Hooft, 2004 ▶); cell refinement: *SCALEPACK* (Otwinowski & Minor, 1997 ▶); data reduction: *DENZO* (Otwinowski & Minor, 1997 ▶) and *SCALEPACK*; program(s) used to solve structure: *SIR97* (Altomare *et al.*, 1999 ▶); program(s) used to refine structure: *SHELXL97* (Sheldrick, 2008 ▶); molecular graphics: *ORTEP-3* (Farrugia, 1997 ▶) and *OLEX2* (Dolomanov *et al.*, 2004 ▶); software used to prepare material for publication: *PLATON* (Spek, 2009 ▶).

## Supplementary Material

Crystal structure: contains datablock(s) I, global. DOI: 10.1107/S1600536812002826/ld2042sup1.cif

Structure factors: contains datablock(s) I. DOI: 10.1107/S1600536812002826/ld2042Isup2.hkl

Supplementary material file. DOI: 10.1107/S1600536812002826/ld2042Isup3.cml

Additional supplementary materials:  crystallographic information; 3D view; checkCIF report

## Figures and Tables

**Fig. 1.**
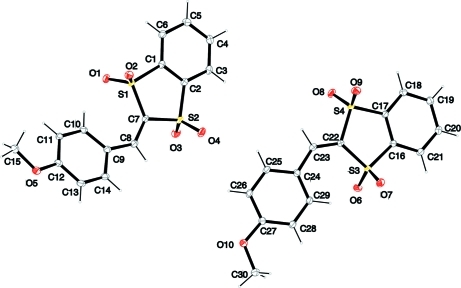
The molecular structures of the two symmetrically independent molecules of the title compound, with atom labels and anisotropic displacement ellipsoids (drawn at 50% probability level) for non-H atoms.

**Fig. 2.**
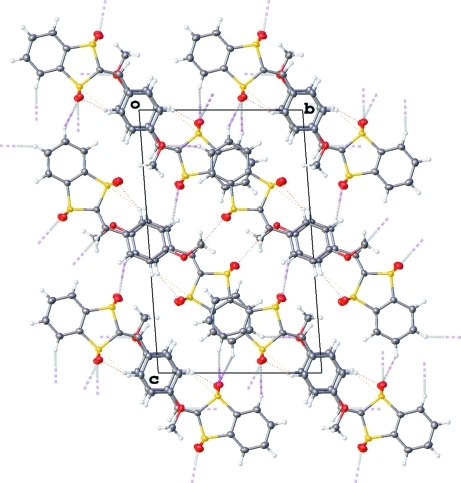
The packing of the title compound viewed along [100].

## supplementary crystallographic information

### Comment

Bissulfonyl ethylenes are important reagents in synthetic organic chemistry, because they are active Michael acceptors (Simpkins, 1993; Najera & Yus, 1999; Prilezhaeva, 2000). Recently, organocatalytic Michael additions of bissulfonyl ethylene have also been reported (Nielsen *et al.*, 2010; Zhu & Lu, 2009; Alba *et al.*, 2010). During our studies on the electrophilic reactivity of bissulfonyl ethylenes, we discussed structure–reactivity relationships.

The asymmetric unit contains two molecules of the title compound (see Fig. 1). In both molecules, the 1,3-benzodithiole plane and the aryl ring of the methoxy-benzylidene group deviate significantly from coplanarity with dihedral angles of 20.41 (9)° and 18.00 (10)°. The dominating feature of the packing of the title compound is π-stacking. The sulfur-bound phenyl rings of the two symmetrically independent molecules are stacked along [100] as well as the anisyl groups of symmetrically dependent molecules (see Fig. 2). This results in three different types of stacks along [100]: (1) two identical stacks made of alternating symmetrically independent sulfur-bound phenyl rings, (2) a stack made of anisyl rings C9···C14 and (3) a stack made of anisyl rings C24···C29. The dihedral angle between the sulfur-bound phenyl rings is 2.15 (13)° with centroid–centroid distances of 3.6659 (16) Å and 3.7288 (16) Å. The π-stackings of the anisyl groups are centrosymmetric that results in coplanar rings. The centroid–centroid distances for the rings C9···C14 are 3.5440 (14) Å and 3.8421 (14), the centroid–centroid distances for C24···C29 are 3.5839 (16) Å and 3.8165 (16) Å. No π-stacking was observed in the related structures (Giacometti *et al.*, 1994; Zhang *et al.*, 2010). The molecules are linked by weak C—H···O interactions with H···O separations shortened up to 2.36 Å. These are indicated by dashed lines in Fig. 2.

### Experimental

4-Methoxybenzylidene 1,3-benzodithiole 1,1,3,3-tetraoxide was synthesized by mixing *p*-anisaldehyde (1.5 g, 11.0 mmol, 6.1 equiv.), 1,3-benzodithiole 1,1,3,3-tetraoxide (400 mg, 1.8 mmol, 1.0 equiv.), diethylammonium chloride (3.4 mmol, 1.9 equiv.) and potassium fluoride (0.27 mmol, 0.15 equiv.) in dry toluene (25 ml) at reflux condition under a Dean Stark water separator for 24 h. After cooling, the solvent was evaporated and the residue was partitioned between water (20 ml) and CH_2_Cl_2_ (20 ml). The organic phase was separated and the aqueous phase was extracted with CH_2_Cl_2_ (3 × 15 ml). The combined organic layer was dried over Na_2_SO_4_, filtered, concentrated under reduced pressure. The crude mixture was purified by flash column chromatography on silica gel (pentane/ethyl acetate: from 95/5 to 80/20), followed by recrystallization from pentane/chloroform to afford yellow crystals. m.p. 222.9–223.9 °C (yield 470 mg, 1.4 mmol, 77.6%).

### Refinement

C-bound H atoms were positioned geometrically (C—H = 0.98 Å for aliphatic, 0.95 Å for aromatic H) and treated as riding on their parent atoms [*U*_iso_(H) = 1.2*U*_eq_(C, aromatic), *U*_iso_(H) = 1.5*U*_eq_(C, aliphatic)]. The methyl groups were allowed to rotate along the C—O bonds to best fit the experimental electron density.

### Crystal data

**Table:**

C_15_H_12_O_5_S_2_	*Z* = 4
*M**_r_* = 336.39	*F*(000) = 696
Triclinic, *P*1	*D*_x_ = 1.593 (1) Mg m^−^^3^
Hall symbol: -P 1	Mo *K*α radiation, λ = 0.71073 Å
*a* = 7.3649 (2) Å	Cell parameters from 5056 reflections
*b* = 11.4723 (3) Å	θ = 3.1–25.4°
*c* = 17.6114 (5) Å	µ = 0.40 mm^−^^1^
α = 84.345 (2)°	*T* = 173 K
β = 84.631 (2)°	Block, yellow
γ = 71.711 (2)°	0.17 × 0.12 × 0.08 mm
*V* = 1402.89 (7) Å^3^	

### Data collection

**Table:**

Nonius KappaCCD diffractometer	4435 reflections with *I* > 2σ(*I*)
Radiation source: rotating anode	*R*_int_ = 0.019
MONTEL, graded multilayered X-ray optics monochromator	θ_max_ = 25.4°, θ_min_ = 3.3°
CCD; rotation images; thick slices scans	*h* = −8→8
9496 measured reflections	*k* = −13→13
5124 independent reflections	*l* = −20→21

### Refinement

**Table:**

Refinement on *F*^2^	Primary atom site location: structure-invariant direct methods
Least-squares matrix: full	Secondary atom site location: difference Fourier map
*R*[*F*^2^ > 2σ(*F*^2^)] = 0.037	Hydrogen site location: inferred from neighbouring sites
*wR*(*F*^2^) = 0.093	H-atom parameters constrained
*S* = 1.10	*w* = 1/[σ^2^(*F*_o_^2^) + (0.025*P*)^2^ + 1.7791*P*] where *P* = (*F*_o_^2^ + 2*F*_c_^2^)/3
5124 reflections	(Δ/σ)_max_ = 0.001
399 parameters	Δρ_max_ = 0.29 e Å^−^^3^
0 restraints	Δρ_min_ = −0.38 e Å^−^^3^

### Special details

**Table:**

Geometry. All e.s.d.'s (except the e.s.d. in the dihedral angle between two l.s. planes) are estimated using the full covariance matrix. The cell e.s.d.'s are taken into account individually in the estimation of e.s.d.'s in distances, angles and torsion angles; correlations between e.s.d.'s in cell parameters are only used when they are defined by crystal symmetry. An approximate (isotropic) treatment of cell e.s.d.'s is used for estimating e.s.d.'s involving l.s. planes.
Refinement. Refinement of *F*^2^ against ALL reflections. The weighted *R*-factor *wR* and goodness of fit *S* are based on *F*^2^, conventional *R*-factors *R* are based on *F*, with *F* set to zero for negative *F*^2^. The threshold expression of *F*^2^ > 2*σ*(*F*^2^) is used only for calculating *R*-factors(gt) *etc*. and is not relevant to the choice of reflections for refinement. *R*-factors based on *F*^2^ are statistically about twice as large as those based on *F*, and *R*-factors based on all data will be even larger.

### Fractional atomic coordinates and isotropic or equivalent isotropic displacement parameters (Å^2^)

**Table:**

	*x*	*y*	*z*	*U*_iso_*/*U*_eq_	
S1	0.64347 (9)	0.35339 (5)	0.08928 (3)	0.02238 (14)	
S2	0.64751 (9)	0.25433 (6)	0.24987 (3)	0.02422 (15)	
O1	0.8215 (3)	0.35541 (16)	0.04909 (10)	0.0295 (4)	
O2	0.4828 (3)	0.36877 (16)	0.04470 (9)	0.0289 (4)	
O3	0.8294 (3)	0.21116 (17)	0.28301 (10)	0.0323 (4)	
O4	0.4931 (3)	0.21619 (17)	0.28849 (10)	0.0336 (4)	
O5	0.7530 (3)	−0.12438 (15)	−0.13537 (10)	0.0314 (4)	
C1	0.5826 (3)	0.4621 (2)	0.15891 (13)	0.0232 (5)	
C2	0.5810 (3)	0.4154 (2)	0.23440 (13)	0.0233 (5)	
C3	0.5398 (4)	0.4931 (2)	0.29365 (14)	0.0280 (5)	
H3	0.5384	0.4613	0.3455	0.034*	
C4	0.5008 (4)	0.6182 (2)	0.27511 (15)	0.0301 (6)	
H4	0.4718	0.6731	0.3147	0.036*	
C5	0.5035 (4)	0.6643 (2)	0.19921 (15)	0.0297 (6)	
H5	0.4773	0.7504	0.1877	0.036*	
C6	0.5439 (4)	0.5870 (2)	0.14008 (14)	0.0268 (5)	
H6	0.5450	0.6188	0.0882	0.032*	
C7	0.6748 (3)	0.2207 (2)	0.15259 (13)	0.0222 (5)	
C8	0.7040 (3)	0.1042 (2)	0.13583 (14)	0.0248 (5)	
H8	0.7087	0.0476	0.1793	0.030*	
C9	0.7299 (3)	0.0493 (2)	0.06317 (14)	0.0237 (5)	
C10	0.7700 (3)	0.1060 (2)	−0.00806 (14)	0.0241 (5)	
H10	0.7893	0.1844	−0.0101	0.029*	
C11	0.7821 (3)	0.0502 (2)	−0.07497 (14)	0.0237 (5)	
H11	0.8092	0.0900	−0.1226	0.028*	
C12	0.7542 (3)	−0.0654 (2)	−0.07256 (14)	0.0250 (5)	
C13	0.7247 (3)	−0.1262 (2)	−0.00221 (15)	0.0273 (5)	
H13	0.7134	−0.2067	0.0000	0.033*	
C14	0.7122 (3)	−0.0693 (2)	0.06378 (15)	0.0262 (5)	
H14	0.6909	−0.1113	0.1114	0.031*	
C15	0.7570 (4)	−0.0591 (2)	−0.20891 (15)	0.0328 (6)	
H15A	0.8792	−0.0413	−0.2187	0.049*	
H15B	0.6512	0.0182	−0.2099	0.049*	
H15C	0.7434	−0.1097	−0.2484	0.049*	
S3	0.06712 (9)	0.22772 (5)	0.71282 (3)	0.02390 (15)	
S4	0.00344 (9)	0.46523 (5)	0.62306 (3)	0.02375 (15)	
O6	0.2566 (3)	0.15179 (16)	0.73049 (10)	0.0338 (4)	
O7	−0.0825 (3)	0.17105 (16)	0.72510 (10)	0.0349 (4)	
O8	0.1567 (3)	0.51075 (16)	0.59109 (10)	0.0331 (4)	
O9	−0.1808 (3)	0.52166 (16)	0.59230 (10)	0.0336 (4)	
O10	0.3914 (3)	−0.20996 (16)	0.44004 (10)	0.0325 (4)	
C16	0.0027 (3)	0.3599 (2)	0.76490 (13)	0.0218 (5)	
C17	−0.0216 (3)	0.4715 (2)	0.72270 (13)	0.0218 (5)	
C18	−0.0697 (3)	0.5813 (2)	0.75786 (14)	0.0253 (5)	
H18	−0.0866	0.6576	0.7285	0.030*	
C19	−0.0923 (4)	0.5763 (2)	0.83682 (14)	0.0277 (5)	
H19	−0.1233	0.6499	0.8623	0.033*	
C20	−0.0701 (4)	0.4644 (2)	0.87937 (14)	0.0284 (5)	
H20	−0.0880	0.4630	0.9335	0.034*	
C21	−0.0226 (4)	0.3551 (2)	0.84416 (14)	0.0264 (5)	
H21	−0.0077	0.2790	0.8734	0.032*	
C22	0.0720 (3)	0.3032 (2)	0.62085 (13)	0.0226 (5)	
C23	0.1276 (3)	0.2572 (2)	0.55205 (14)	0.0252 (5)	
H23	0.1287	0.3184	0.5117	0.030*	
C24	0.1858 (3)	0.1340 (2)	0.52707 (13)	0.0234 (5)	
C25	0.2688 (4)	0.1172 (2)	0.45215 (13)	0.0261 (5)	
H25	0.2797	0.1866	0.4200	0.031*	
C26	0.3345 (4)	0.0027 (2)	0.42451 (14)	0.0267 (5)	
H26	0.3914	−0.0070	0.3739	0.032*	
C27	0.3171 (4)	−0.0996 (2)	0.47123 (14)	0.0247 (5)	
C28	0.2291 (3)	−0.0849 (2)	0.54467 (13)	0.0247 (5)	
H28	0.2131	−0.1540	0.5757	0.030*	
C29	0.1654 (4)	0.0303 (2)	0.57202 (14)	0.0249 (5)	
H29	0.1065	0.0398	0.6223	0.030*	
C30	0.3957 (5)	−0.3186 (2)	0.48879 (16)	0.0379 (7)	
H30A	0.4664	−0.3210	0.5337	0.057*	
H30B	0.4592	−0.3917	0.4606	0.057*	
H30C	0.2643	−0.3174	0.5054	0.057*	

### Atomic displacement parameters (Å^2^)

**Table:**

	*U*^11^	*U*^22^	*U*^33^	*U*^12^	*U*^13^	*U*^23^
S1	0.0289 (3)	0.0221 (3)	0.0162 (3)	−0.0091 (2)	−0.0007 (2)	0.0013 (2)
S2	0.0285 (3)	0.0270 (3)	0.0170 (3)	−0.0099 (3)	−0.0010 (2)	0.0032 (2)
O1	0.0348 (10)	0.0310 (10)	0.0238 (9)	−0.0143 (8)	0.0070 (8)	−0.0020 (7)
O2	0.0352 (10)	0.0293 (9)	0.0216 (9)	−0.0074 (8)	−0.0091 (7)	−0.0008 (7)
O3	0.0334 (10)	0.0374 (10)	0.0236 (9)	−0.0074 (8)	−0.0087 (8)	0.0049 (8)
O4	0.0381 (11)	0.0392 (11)	0.0262 (9)	−0.0197 (9)	0.0068 (8)	0.0018 (8)
O5	0.0418 (11)	0.0221 (9)	0.0313 (10)	−0.0111 (8)	0.0000 (8)	−0.0051 (7)
C1	0.0221 (12)	0.0296 (13)	0.0186 (12)	−0.0093 (10)	−0.0020 (9)	−0.0005 (10)
C2	0.0221 (12)	0.0279 (13)	0.0210 (12)	−0.0098 (10)	−0.0017 (9)	0.0011 (10)
C3	0.0313 (14)	0.0360 (14)	0.0180 (12)	−0.0124 (11)	−0.0007 (10)	−0.0024 (10)
C4	0.0285 (14)	0.0353 (15)	0.0255 (13)	−0.0071 (11)	0.0003 (11)	−0.0086 (11)
C5	0.0319 (14)	0.0246 (13)	0.0321 (14)	−0.0075 (11)	−0.0047 (11)	−0.0008 (11)
C6	0.0311 (14)	0.0281 (13)	0.0211 (12)	−0.0092 (11)	−0.0035 (10)	0.0012 (10)
C7	0.0236 (12)	0.0254 (12)	0.0176 (11)	−0.0088 (10)	−0.0011 (9)	0.0024 (9)
C8	0.0226 (12)	0.0263 (13)	0.0241 (12)	−0.0076 (10)	−0.0014 (10)	0.0043 (10)
C9	0.0202 (12)	0.0228 (12)	0.0270 (13)	−0.0056 (10)	−0.0022 (10)	0.0010 (10)
C10	0.0211 (12)	0.0202 (12)	0.0308 (13)	−0.0066 (10)	−0.0005 (10)	−0.0011 (10)
C11	0.0217 (12)	0.0224 (12)	0.0265 (13)	−0.0076 (10)	0.0014 (10)	0.0001 (10)
C12	0.0217 (12)	0.0201 (12)	0.0320 (14)	−0.0045 (10)	−0.0011 (10)	−0.0035 (10)
C13	0.0246 (13)	0.0181 (12)	0.0379 (15)	−0.0056 (10)	−0.0017 (11)	0.0008 (10)
C14	0.0220 (12)	0.0216 (12)	0.0319 (14)	−0.0043 (10)	−0.0019 (10)	0.0043 (10)
C15	0.0423 (16)	0.0279 (14)	0.0289 (14)	−0.0114 (12)	−0.0007 (12)	−0.0054 (11)
S3	0.0316 (3)	0.0185 (3)	0.0196 (3)	−0.0067 (2)	0.0012 (2)	0.0023 (2)
S4	0.0327 (3)	0.0194 (3)	0.0186 (3)	−0.0085 (2)	−0.0012 (2)	0.0021 (2)
O6	0.0394 (11)	0.0259 (9)	0.0274 (10)	0.0017 (8)	−0.0043 (8)	0.0032 (7)
O7	0.0465 (12)	0.0287 (10)	0.0340 (10)	−0.0215 (9)	0.0108 (9)	−0.0037 (8)
O8	0.0471 (12)	0.0310 (10)	0.0262 (9)	−0.0223 (9)	0.0083 (8)	−0.0012 (8)
O9	0.0401 (11)	0.0290 (10)	0.0271 (10)	−0.0034 (8)	−0.0110 (8)	0.0034 (8)
O10	0.0474 (11)	0.0234 (9)	0.0250 (9)	−0.0096 (8)	0.0036 (8)	−0.0035 (7)
C16	0.0196 (12)	0.0212 (12)	0.0238 (12)	−0.0061 (9)	0.0014 (9)	−0.0007 (9)
C17	0.0217 (12)	0.0227 (12)	0.0204 (12)	−0.0072 (10)	−0.0002 (9)	0.0007 (9)
C18	0.0279 (13)	0.0213 (12)	0.0276 (13)	−0.0094 (10)	−0.0006 (10)	−0.0004 (10)
C19	0.0279 (13)	0.0283 (13)	0.0281 (13)	−0.0099 (11)	0.0020 (10)	−0.0070 (10)
C20	0.0287 (13)	0.0337 (14)	0.0221 (13)	−0.0098 (11)	0.0026 (10)	−0.0027 (10)
C21	0.0285 (13)	0.0261 (13)	0.0225 (12)	−0.0072 (10)	−0.0003 (10)	0.0035 (10)
C22	0.0271 (13)	0.0187 (12)	0.0209 (12)	−0.0069 (10)	−0.0002 (10)	0.0020 (9)
C23	0.0276 (13)	0.0264 (13)	0.0211 (12)	−0.0094 (10)	−0.0031 (10)	0.0048 (10)
C24	0.0241 (12)	0.0239 (12)	0.0219 (12)	−0.0064 (10)	−0.0025 (10)	−0.0023 (10)
C25	0.0334 (14)	0.0279 (13)	0.0181 (12)	−0.0126 (11)	−0.0024 (10)	0.0039 (10)
C26	0.0335 (14)	0.0313 (13)	0.0159 (11)	−0.0118 (11)	0.0000 (10)	−0.0005 (10)
C27	0.0284 (13)	0.0248 (13)	0.0214 (12)	−0.0083 (10)	−0.0036 (10)	−0.0029 (10)
C28	0.0287 (13)	0.0257 (13)	0.0201 (12)	−0.0100 (10)	−0.0007 (10)	0.0013 (10)
C29	0.0276 (13)	0.0282 (13)	0.0184 (12)	−0.0091 (10)	0.0009 (10)	−0.0001 (10)
C30	0.0569 (19)	0.0241 (14)	0.0305 (14)	−0.0117 (13)	0.0034 (13)	−0.0003 (11)

### Geometric parameters (Å, º)

**Table:**

S1—O1	1.4360 (18)	S3—O7	1.4353 (18)
S1—O2	1.4362 (18)	S3—O6	1.4380 (19)
S1—C1	1.763 (2)	S3—C22	1.763 (2)
S1—C7	1.763 (2)	S3—C16	1.763 (2)
S2—O3	1.4328 (18)	S4—O8	1.4343 (18)
S2—O4	1.4342 (18)	S4—O9	1.4389 (19)
S2—C2	1.757 (2)	S4—C17	1.754 (2)
S2—C7	1.772 (2)	S4—C22	1.770 (2)
O5—C12	1.354 (3)	O10—C27	1.360 (3)
O5—C15	1.433 (3)	O10—C30	1.436 (3)
C1—C6	1.383 (3)	C16—C17	1.385 (3)
C1—C2	1.384 (3)	C16—C21	1.389 (3)
C2—C3	1.390 (3)	C17—C18	1.388 (3)
C3—C4	1.384 (4)	C18—C19	1.383 (4)
C3—H3	0.9500	C18—H18	0.9500
C4—C5	1.389 (4)	C19—C20	1.392 (4)
C4—H4	0.9500	C19—H19	0.9500
C5—C6	1.385 (4)	C20—C21	1.384 (4)
C5—H5	0.9500	C20—H20	0.9500
C6—H6	0.9500	C21—H21	0.9500
C7—C8	1.343 (3)	C22—C23	1.346 (3)
C8—C9	1.452 (3)	C23—C24	1.442 (3)
C8—H8	0.9500	C23—H23	0.9500
C9—C14	1.405 (3)	C24—C29	1.404 (3)
C9—C10	1.405 (3)	C24—C25	1.406 (3)
C10—C11	1.378 (3)	C25—C26	1.371 (4)
C10—H10	0.9500	C25—H25	0.9500
C11—C12	1.400 (3)	C26—C27	1.398 (3)
C11—H11	0.9500	C26—H26	0.9500
C12—C13	1.394 (4)	C27—C28	1.393 (3)
C13—C14	1.370 (4)	C28—C29	1.376 (3)
C13—H13	0.9500	C28—H28	0.9500
C14—H14	0.9500	C29—H29	0.9500
C15—H15A	0.9800	C30—H30A	0.9800
C15—H15B	0.9800	C30—H30B	0.9800
C15—H15C	0.9800	C30—H30C	0.9800
			
O1—S1—O2	117.71 (11)	O7—S3—O6	116.77 (12)
O1—S1—C1	108.95 (11)	O7—S3—C22	112.52 (11)
O2—S1—C1	110.29 (11)	O6—S3—C22	110.41 (11)
O1—S1—C7	111.23 (11)	O7—S3—C16	108.99 (11)
O2—S1—C7	109.43 (11)	O6—S3—C16	109.12 (11)
C1—S1—C7	97.34 (11)	C22—S3—C16	97.21 (11)
O3—S2—O4	117.61 (11)	O8—S4—O9	116.81 (11)
O3—S2—C2	108.98 (11)	O8—S4—C17	110.52 (11)
O4—S2—C2	110.83 (11)	O9—S4—C17	109.14 (11)
O3—S2—C7	110.16 (11)	O8—S4—C22	110.39 (11)
O4—S2—C7	110.04 (11)	O9—S4—C22	110.62 (11)
C2—S2—C7	97.32 (11)	C17—S4—C22	97.69 (11)
C12—O5—C15	118.30 (19)	C27—O10—C30	117.44 (19)
C6—C1—C2	121.2 (2)	C17—C16—C21	120.5 (2)
C6—C1—S1	122.46 (18)	C17—C16—S3	116.57 (18)
C2—C1—S1	116.29 (19)	C21—C16—S3	122.91 (18)
C1—C2—C3	120.8 (2)	C16—C17—C18	121.4 (2)
C1—C2—S2	116.23 (18)	C16—C17—S4	115.80 (18)
C3—C2—S2	122.88 (19)	C18—C17—S4	122.71 (18)
C4—C3—C2	118.2 (2)	C19—C18—C17	118.0 (2)
C4—C3—H3	120.9	C19—C18—H18	121.0
C2—C3—H3	120.9	C17—C18—H18	121.0
C3—C4—C5	120.7 (2)	C18—C19—C20	120.7 (2)
C3—C4—H4	119.7	C18—C19—H19	119.6
C5—C4—H4	119.7	C20—C19—H19	119.6
C6—C5—C4	121.2 (2)	C21—C20—C19	121.2 (2)
C6—C5—H5	119.4	C21—C20—H20	119.4
C4—C5—H5	119.4	C19—C20—H20	119.4
C1—C6—C5	117.9 (2)	C20—C21—C16	118.2 (2)
C1—C6—H6	121.0	C20—C21—H21	120.9
C5—C6—H6	121.0	C16—C21—H21	120.9
C8—C7—S1	128.34 (19)	C23—C22—S3	130.49 (19)
C8—C7—S2	118.91 (18)	C23—C22—S4	116.88 (18)
S1—C7—S2	112.62 (13)	S3—C22—S4	112.52 (13)
C7—C8—C9	131.6 (2)	C22—C23—C24	133.1 (2)
C7—C8—H8	114.2	C22—C23—H23	113.4
C9—C8—H8	114.2	C24—C23—H23	113.4
C14—C9—C10	117.3 (2)	C29—C24—C25	117.9 (2)
C14—C9—C8	117.7 (2)	C29—C24—C23	124.8 (2)
C10—C9—C8	125.0 (2)	C25—C24—C23	117.3 (2)
C11—C10—C9	121.3 (2)	C26—C25—C24	121.3 (2)
C11—C10—H10	119.3	C26—C25—H25	119.3
C9—C10—H10	119.3	C24—C25—H25	119.3
C10—C11—C12	119.8 (2)	C25—C26—C27	119.6 (2)
C10—C11—H11	120.1	C25—C26—H26	120.2
C12—C11—H11	120.1	C27—C26—H26	120.2
O5—C12—C13	116.2 (2)	O10—C27—C28	124.1 (2)
O5—C12—C11	124.1 (2)	O10—C27—C26	115.6 (2)
C13—C12—C11	119.7 (2)	C28—C27—C26	120.2 (2)
C14—C13—C12	119.7 (2)	C29—C28—C27	119.6 (2)
C14—C13—H13	120.1	C29—C28—H28	120.2
C12—C13—H13	120.1	C27—C28—H28	120.2
C13—C14—C9	122.0 (2)	C28—C29—C24	121.3 (2)
C13—C14—H14	119.0	C28—C29—H29	119.3
C9—C14—H14	119.0	C24—C29—H29	119.3
O5—C15—H15A	109.5	O10—C30—H30A	109.5
O5—C15—H15B	109.5	O10—C30—H30B	109.5
H15A—C15—H15B	109.5	H30A—C30—H30B	109.5
O5—C15—H15C	109.5	O10—C30—H30C	109.5
H15A—C15—H15C	109.5	H30A—C30—H30C	109.5
H15B—C15—H15C	109.5	H30B—C30—H30C	109.5
			
O1—S1—C1—C6	63.6 (2)	O7—S3—C16—C17	−117.07 (19)
O2—S1—C1—C6	−67.1 (2)	O6—S3—C16—C17	114.37 (19)
C7—S1—C1—C6	179.0 (2)	C22—S3—C16—C17	−0.2 (2)
O1—S1—C1—C2	−114.48 (19)	O7—S3—C16—C21	63.2 (2)
O2—S1—C1—C2	114.90 (19)	O6—S3—C16—C21	−65.4 (2)
C7—S1—C1—C2	1.0 (2)	C22—S3—C16—C21	−180.0 (2)
C6—C1—C2—C3	0.2 (4)	C21—C16—C17—C18	0.7 (4)
S1—C1—C2—C3	178.31 (18)	S3—C16—C17—C18	−179.10 (18)
C6—C1—C2—S2	−176.12 (19)	C21—C16—C17—S4	−177.07 (19)
S1—C1—C2—S2	2.0 (3)	S3—C16—C17—S4	3.2 (3)
O3—S2—C2—C1	110.47 (19)	O8—S4—C17—C16	−119.63 (19)
O4—S2—C2—C1	−118.59 (19)	O9—S4—C17—C16	110.60 (19)
C7—S2—C2—C1	−3.8 (2)	C22—S4—C17—C16	−4.4 (2)
O3—S2—C2—C3	−65.8 (2)	O8—S4—C17—C18	62.7 (2)
O4—S2—C2—C3	65.1 (2)	O9—S4—C17—C18	−67.1 (2)
C7—S2—C2—C3	179.9 (2)	C22—S4—C17—C18	177.9 (2)
C1—C2—C3—C4	−0.2 (4)	C16—C17—C18—C19	0.2 (4)
S2—C2—C3—C4	175.95 (19)	S4—C17—C18—C19	177.78 (19)
C2—C3—C4—C5	−0.2 (4)	C17—C18—C19—C20	−1.0 (4)
C3—C4—C5—C6	0.5 (4)	C18—C19—C20—C21	0.8 (4)
C2—C1—C6—C5	0.0 (4)	C19—C20—C21—C16	0.0 (4)
S1—C1—C6—C5	−177.93 (19)	C17—C16—C21—C20	−0.8 (4)
C4—C5—C6—C1	−0.4 (4)	S3—C16—C21—C20	178.96 (19)
O1—S1—C7—C8	−74.0 (2)	O7—S3—C22—C23	−72.5 (3)
O2—S1—C7—C8	57.7 (3)	O6—S3—C22—C23	60.0 (3)
C1—S1—C7—C8	172.3 (2)	C16—S3—C22—C23	173.5 (2)
O1—S1—C7—S2	110.16 (14)	O7—S3—C22—S4	111.31 (14)
O2—S1—C7—S2	−118.08 (13)	O6—S3—C22—S4	−116.27 (14)
C1—S1—C7—S2	−3.49 (15)	C16—S3—C22—S4	−2.74 (15)
O3—S2—C7—C8	74.7 (2)	O8—S4—C22—C23	−57.4 (2)
O4—S2—C7—C8	−56.5 (2)	O9—S4—C22—C23	73.5 (2)
C2—S2—C7—C8	−171.9 (2)	C17—S4—C22—C23	−172.7 (2)
O3—S2—C7—S1	−109.02 (14)	O8—S4—C22—S3	119.43 (14)
O4—S2—C7—S1	119.71 (13)	O9—S4—C22—S3	−109.73 (14)
C2—S2—C7—S1	4.33 (15)	C17—S4—C22—S3	4.12 (15)
S1—C7—C8—C9	4.0 (4)	S3—C22—C23—C24	6.4 (4)
S2—C7—C8—C9	179.6 (2)	S4—C22—C23—C24	−177.5 (2)
C7—C8—C9—C14	−165.1 (3)	C22—C23—C24—C29	11.6 (4)
C7—C8—C9—C10	14.5 (4)	C22—C23—C24—C25	−168.5 (3)
C14—C9—C10—C11	3.2 (3)	C29—C24—C25—C26	−2.3 (4)
C8—C9—C10—C11	−176.4 (2)	C23—C24—C25—C26	177.7 (2)
C9—C10—C11—C12	−0.1 (4)	C24—C25—C26—C27	0.6 (4)
C15—O5—C12—C13	171.8 (2)	C30—O10—C27—C28	−6.4 (4)
C15—O5—C12—C11	−7.9 (3)	C30—O10—C27—C26	173.3 (2)
C10—C11—C12—O5	176.3 (2)	C25—C26—C27—O10	−178.0 (2)
C10—C11—C12—C13	−3.4 (4)	C25—C26—C27—C28	1.7 (4)
O5—C12—C13—C14	−176.0 (2)	O10—C27—C28—C29	177.4 (2)
C11—C12—C13—C14	3.7 (4)	C26—C27—C28—C29	−2.3 (4)
C12—C13—C14—C9	−0.6 (4)	C27—C28—C29—C24	0.6 (4)
C10—C9—C14—C13	−2.9 (4)	C25—C24—C29—C28	1.7 (4)
C8—C9—C14—C13	176.7 (2)	C23—C24—C29—C28	−178.3 (2)

### Hydrogen-bond geometry (Å, º)

**Table:**

*D*—H···*A*	*D*—H	H···*A*	*D*···*A*	*D*—H···*A*
C6—H6···O2^i^	0.95	2.36	3.259 (3)	159
C19—H19···O5^ii^	0.95	2.46	3.332 (3)	152
C20—H20···O1^iii^	0.95	2.42	3.252 (3)	146
C23—H23···O9^iv^	0.95	2.55	3.496 (3)	172
C28—H28···O3^v^	0.95	2.53	3.282 (3)	137

Symmetry codes: (i) −*x*+1, −*y*+1, −*z*; (ii) *x*−1, *y*+1, *z*+1; (iii) *x*−1, *y*, *z*+1; (iv) −*x*, −*y*+1, −*z*+1; (v) −*x*+1, −*y*, −*z*+1.
